# Biogenic Amine Levels Markedly Increase in the Aqueous Humor of Individuals with Controlled Type 2 Diabetes

**DOI:** 10.3390/ijms232112752

**Published:** 2022-10-22

**Authors:** Alejandro Lillo, Silvia Marin, Joan Serrano-Marín, David Bernal-Casas, Nicolas Binetti, Gemma Navarro, Marta Cascante, Juan Sánchez-Navés, Rafael Franco

**Affiliations:** 1 Department of Biochemistry and Physiology, School of Pharmacy and Food Science, Universitat de Barcelona, 08028 Barcelona, Spain; 2 CiberNed, Network Center for Neurodegenerative Diseases, National Spanish Health Institute Carlos III, 28029 Madrid, Spain; 3 Department of Biochemistry and Molecular Biomedicine, Faculty of Biology, Universitat de Barcelona, 08028 Barcelona, Spain; 4 Institute of Biomedicine of University of Barcelona (IBUB), University of Barcelona, 08028 Barcelona, Spain; 5 CIBEREHD, Network Center for Hepatic and Digestive Diseases, National Spanish Health Institute Carlos III (ISCIII), 28029 Madrid, Spain; 6 Molecular Neurobiology Laboratory, Department of Biochemistry and Molecular Biomedicine, Universitat de Barcelona, 08028 Barcelona, Spain; 7 Department of Genetics, Microbiology and Statistics, Faculty of Biology, Universitat de Barcelona, 08028 Barcelona, Spain; 8 Department of Ophthalmology, Ophthalmedic and I.P.O, Institute of Ophthalmology, 07011 Palma de Mallorca, Spain; 9 School of Chemistry, Universitat de Barcelona, 08028 Barcelona, Spain

**Keywords:** ornithine, alanine, metabolomics, glutamine, biogenic amines, type 2 diabetes, retinopathy, mass spectrometry, NAT8, aqueous humor

## Abstract

The composition of the aqueous humor of patients with type 2 diabetes is relevant to understanding the underlying causes of eye-related comorbidities. Information on the composition of aqueous humor in healthy subjects is limited due to the lack of adequate controls. To carry out a metabolomics study, 31 samples of aqueous humor from healthy subjects without ocular pathology, submitted to refractive surgery and seven samples from patients with type 2 diabetes without signs of ocular pathology related to diabetes were used. The level of 25 molecules was significantly (*p* < 0.001) altered in the aqueous humor of the patient group. The concentration of a single molecule, N-acetylornithine, makes it possible to discriminate between control and diabetes (sensitivity and specificity equal to 1). In addition, receptor operating characteristic curve and principal component analysis for the above-mentioned six molecules yielded significantly (*p* < 0.001) altered in the aqueous humor of the patient group. In addition, receptor operating characteristic curve and principal component analysis for six compounds yielded cut-off values and remarkable sensitivity, specificity, and segregation ability. The altered level of N-acetylornithine may be due to an increased amount of acetate in diabetes. It is of interest to further investigate whether this alteration is related to the pathogenesis of the disease. The increase in the amino form of pyruvate, alanine, in diabetes is also relevant because it could be a means of reducing the formation of lactate from pyruvate.

## 1. Introduction

Diabetes is a disorder of glucose metabolism and handling that can manifest in early childhood (type 1 diabetes) or later in life (mainly type 2 diabetes) [1,2,3]. Type 2 diabetes is associated with metabolic diseases, obesity, and/or sedentary lifestyle. Actual data has exceeded projections, such that the prevalence of type 2 diabetes in youth has doubled in 12 to 16 years in both the United States and China [4,5]. This means that the number of years a patient will live with disease is longer than ever, leading to an increase in the number and severity of accompanying clinical symptoms.

According to the World Health Organization “Diabetes is a major cause of blindness, kidney failure, heart attacks, stroke and lower limb amputation” (https://www.who.int/news-room/fact-sheets/detail/diabetes, accessed on 9 July 2022). Ocular alterations cause discomfort and significantly reduce the quality of life of diabetic patients; as an example, the prevalence of dry-eye syndrome is >50% in type 2 patients [6]. Another concern derives from the neurological alterations associated with diabetes that affect cognitive functions, but also sensory and motor control functions [7]. The neurological manifestations are due to functional and structural alterations, some of which present variable degrees of microglial activation [8,9,10,11]. For ethical reasons derived from the invasiveness of obtaining it, cerebrospinal fluid (CSF) has not been extensively studied in diabetic patients. An alternative is aqueous humor (AH), a body fluid whose composition apparently varies with that of plasma and CSF. Indeed, there is evidence of fluid exchange between CSF and AH [12,13].

AH provides nutrients to the cornea and lens; by maintaining intraocular pressure, it preserves the three-dimensional structure of the oculus and facilitates the focusing of images on the retina. Yudkin [14] provided the first account on the formation and chemical composition of aqueous humor, which must be transparent and have an appropriate refraction index. Apart from proteins and other macromolecules (e.g., miRNA) this body fluid contains molecules as diverse as lipids, amino acids, vitamins, and intermediary metabolites [15,16,17] and fills the anterior and posterior chambers of the eye.

The aim of this study was to compare the composition of the AH of type 2 diabetic patients and healthy controls based on the determination of the concentration of 188 metabolites, from total sugars, to acyl-carnitines, sphingomyelins, lysophosphatidylcholines, amino acids, and biogenic amines (BAs).

## 2. Results

**Figure 1:**
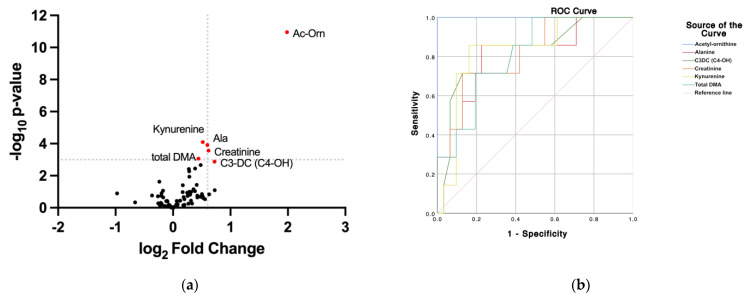
(**a**) Volcano plot (statistical significance versus log_2_ fold change) upon comparing data from control and diabetes groups. Red color indicates data points for molecules whose log_2_ FC > |0.4| and *p* < 0.001. (**b**) ROC curve of the six selected molecules.

Analysis of data from healthy controls and patients diagnosed with type 2 diabetes led to finding reliable concentrations for 80 molecules (all values are provided in Supplementary Table S1). Metabolites with concentration values below the detection limit, or whose values are not within the standard curve, were not included in the analysis. When comparing the data in samples from diabetic patients and healthy controls, the concentration of 25 molecules was significantly different (Supplementary Table S1). The compound whose range of variation between diabetics and controls was greater and whose statistical score was higher was N-acetylornithine (see volcano plot in Figure 1). A receiver operating characteristic curve (ROC) was constructed for N-acetylornithine, whose concentration variation had *p* < 0.001 and log_2_ fold change (FC) > |1|. Moving thresholds to *p* < 0.001 and log_2_ fold change (FC) > |0.4|, five additional molecules were selected: kynurenine, creatinine, dimethylarginines (DMA), hydroxy-butyryl-carnitine (also known as malonyl-carnitine or C4-OH acyl-carnitine), and alanine. Remarkably, the levels of the selected molecules were increased in diabetes.

**Figure 2:**
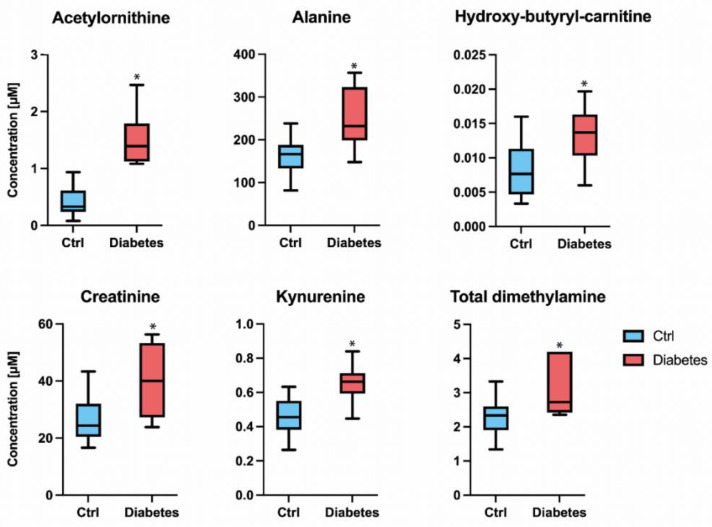
Comparison of the concentration of N-acetylornithine, alanine, hydroxy-butyryl-carnitine, kynurenine, creatinine, and total DMA in the aqueous humor of the two groups. Median in box-and-whisker plots (whiskers indicate the highest and lowest values determined for every molecule in each group, control, or diabetes). * *p*-value < 0.001.

The area under ROC (AUC) for N-acetylornithine was 1 and the sensitivity and specificity were 1, that is, the measurement of the level of N-acetylornithine in the aqueous humor would allow to distinguish between healthy controls and diabetic patients. The AUC for the other five molecules was high, close to 0.8, with remarkable sensitivity and specificity values (Table 1); consequently, the cut-off values in Table 1 seem reliable. The difference in concentration between the control and diabetes groups is shown in Figure 2 for N-acetylornithine, kynurenine, creatinine, total dimethylarginines (DMA), hydroxy-butyryl-carnitine, and alanine. There is a wide difference in the concentration of N-acetylornithine between AH in controls and in type 2 diabetes patients; therefore, the specificity is maximum even with cut-off value higher than that in Table 1, 1.01 µM. This means that there would be little risk of false positives, using the concentration of this molecule as a parameter. Correlation analysis of the concentration values of these metabolites in controls and diabetes showed several significant correlation coefficients for creatinine and total DMA (r = 0.77), for alanine and total DMA (r = 0.76) and for alanine and creatinine (r = 0.74). It should be noted that all the pairwise correlations between the BAs were significant and that the correlations of N-acetylornithine with the other five molecules were also significant (Table 2). Principal component analysis for the six selected molecules shows good separation between cases and controls (Supplementary Figure S1).

## 3. Discussion

Metabolomics is becoming essential to understanding the composition of the aqueous humor in both health and disease. Alterations in the concentrations of molecular components in the aqueous humor can provide relevant information to understand whether a certain comorbidity (e.g., cataracts, kidney disease) is the consequence of a chronic disease, such as type 2 diabetes.

We present here complementary information related to the composition of AH samples from healthy individuals without known eye disease. In a previous study we could analyze only 16 samples from healthy individuals and, in this study, we have incorporated data from 15 more healthy individuals. The median value of all molecules whose concentration can be reliably determined is similar. Adding more controls does not significantly change the findings that were reported in a previous study related to altered AH composition in glaucoma [18]. As in glaucoma and with respect to metabolites whose concentration is significantly altered, the AH of diabetic patients shows much more increase than decrease in the concentration of metabolites. Taking the threshold of *p* < 0.001 and log_2_FC > |1| (FC: fold change), N-acetylornithine is the only molecule that increases more than two-fold (FC = 3.8) in diabetes samples. The median concentration of N-acetylornithine ranges from 0.41 in the control to 1.55 µM in the diabetes samples. When the threshold drops to *p* < 0.001 and log_2_FC > |0.4|, there are five other molecules that increase their concentration in diabetes: kynurenine, creatinine, total dimethylamines, hydroxy-butyryl-carnitine, and alanine. In terms of the absolute value of the change, we consider alanine and creatinine to be relevant; their median concentration increased in diabetes to >70 and >15 µM, respectively. Kynurenine increased from 0.45 in the control to 0.65 µM in the diabetes samples.

Through ROC analysis, the four highest ranked metabolites are BAs (Figure 1 and Table 1). The statistical significance and high degree of correlation within the BA family suggests a metabolic signature. Alanine also showed a highly significant correlation with total DMA and creatinine (Table 2). Taken together, the increased levels of these molecules could be a pattern particular to patients with diabetes. Interestingly, all metabolites over-represented in diabetic patients have at least one nitrogen in their structure, suggesting an imbalance in metabolisms involving nitrogenated molecules. Principal component analysis separates controls from patients using the six selected molecules (Supplementary Figure S1). For the other 19 molecules, the comparison of the concentration in the HA of the two groups is in Supplementary Figure S2.

Increased alanine concentration in AH also occurs in glaucoma, but to a lesser extent [18]. The increase when the general trend of amino acids is to decrease in diabetes may reflect impaired glucose disposition and the underlying metabolic syndrome. A recent report has shown a positive correlation between alanine levels in serum and insulin resistance in non-diabetic individuals [19]. In addition, the concentration of glutamate, whose accumulation can produce excitotoxicity in the cells of the ocular structures [20], is not significantly altered, neither in diabetes nor in glaucoma. Interestingly, alanine aminotransferase would reduce glutamate and pyruvate levels to produce alanine and oxaloacetate, an intermediate metabolite relevant to Krebs cycle function. In glaucoma, glutamate buffering occurs through the formation of glutamine, the level of which is not significantly altered in diabetes. Indeed, the pattern of variation in glaucoma, where Gln, various acyl-carnitines, and lysophosphatidylcholines are increased [18], is different from that found in diabetes.

Contrary to what was thought for decades, ocular fluids are not independent of CSF; communication can occur through the optic nerve and the perivascular space of the central retinal artery [21,22,23]. Hence, the study of the composition of AH may lead to finding some biomarkers of neuroinflammation of those recently reported for some neurological diseases [24]. Consistent with the hypothesis of a neuroinflammatory component in diabetes, there was an increase in the level of kynurenine, which is related to microglial activation/neuroinflammation [25,26,27]. Our findings are consistent with the increase in 3-OH-kynurenine in the CSF of an experimental diabetic encephalopathy model in rats [28]. In a previous study, we found that kynurenine was over-represented in the AH of glaucoma patients [18]. Considering that glaucoma also has an inflammatory component that is, at least in part, due to the activation of retinal microglia [29], kynurenine emerges as a promising biomarker for eye alterations and other diseases that have a neuroinflammatory component.

The presence of N-acetylornithine in AH is intriguing because the enzymes that specifically handle this molecule are not expressed in mammals. N-acetylornithine carbamoyltransferase (E.C. 2.1.3.9) uses the molecule to synthesize N-acetyl-L-citrulline, which is then processed to arginine. Additionally, glutamate N-acetyltransferase (E.C. 2.3.1.35) catalyzes the interconversion of glutamate and N-acetyl-L-citrulline to L-ornithine and N-acetyl-glutamate; therefore, N-acetylornithine is relevant in the synthesis of arginine in microorganisms [30] and ornithine in plants [31]. To our knowledge, the role of N-acetylornithine in mammals is not considered relevant; the mammalian enzyme putatively using N-acetylornithine is NAT8, a member of the NAT acetyl transferase family of enzymes. NAT8 and/or NAT8 homologues are found in the genome of all vertebrate animals and appear to be related to the metabolism of xenobiotics. NAT8 and/or NAT8 homologues are found in the genome of all vertebrate animals and seem to be related to the metabolism of xenobiotics [32]. On the one hand, NAT8 variants are related to renal alterations [33]. On the other hand, it is known that renal disorders are among the comorbidities associated with diabetes mellitus [34]. The metabolomic profile of plasma from South Asian Indian men shows an increase of the level of N-acetylornithine in patients with type 2 diabetes-derived nephropathy that is further increased in diabetes-derived nephropathy [35]. In addition, a genome wide-association study (GWAS) assessing the concentration of 308 untargeted metabolites among African Americans from the atherosclerosis risk in communities (ARIC) found a correlation between higher serum levels of and N-acetyl-1-methylhistidine and N-acetylornithine and decreased estimated glomerular filtration rate [36]. These results fit with those of another GWAS in samples from German KORA F4 and the British TwinsUK studies in which, after adjusting for age and gender, there is a correlation between N-acetylornithine levels in plasma and estimated glomerular filtration rate [37]. These studies highlight a dysfunction in the metabolism of nitrogenated molecules in type 2 diabetes, even in the absence of nephropathy. The correlation analysis of the AH data showed significance when comparing the data for N-acetylornithine with those for the other five selected molecules. This fact and the various correlations found for the data of the other five molecules also points to an overall alteration in the metabolism of nitrogenated molecules that is reflected in the composition of AH. It should be noted that a ^1^H-NMR-based metabolomic study using AH shows the data consistent with activation of pathways related to energy production and to alanine, glutamate, and aspartate metabolism in patients with diabetes mellitus (and cataracts) [38]. Additionally, increased N-acetylornithine as a result of increased acetate concentration would be similar to the production of polyols by aldose reductase when glucose accumulates in extracellular body fluids. The enzyme’s affinity for glucose is low, and catalytic activity only becomes significant when glucose availability increases; cataracts associated with diabetes are caused by excess polyol produced by aldose reductase acting on glucose (see Ref. [39] for review). With all this evidence, it is worth considering whether an increase in N-acetylornithine in diabetes can lead to an alteration of ocular structures and functions.

Although a decreased serum creatinine concentration is a risk factor for developing diabetes, once diabetes is established and the kidney has been affected, creatinine levels increase as a consequence of low renal clearance and impaired protein synthesis [40]. Increased serum creatinine concentration is a common finding in diabetic nephropathy. Interestingly, the high levels of creatinine found in the blood and CSF found in these patients [41] were also detected in other body fluids, such as saliva [42]. Here we show that the creatinine level is also high in AH, even in the absence of nephropathy. Furthermore, the level of dimethylamine is elevated in the AH of diabetic patients. This result is consistent with a decrease in renal clearance.

**Figure 3:**
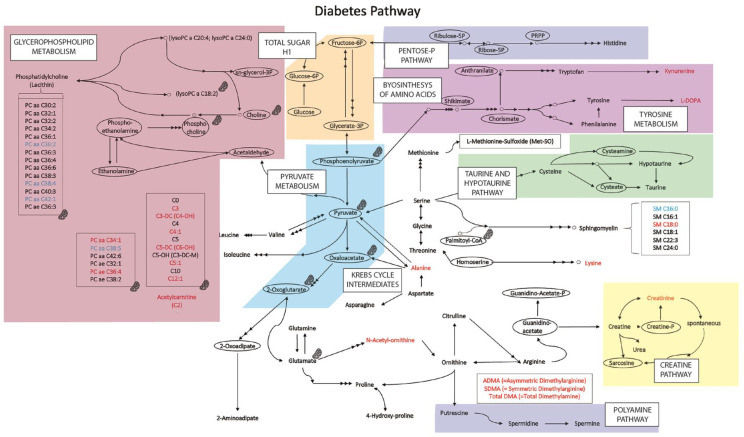
Metabolic pathway map summarizing the results. In light blue, the metabolite whose concentration is reduced in diabetes samples; and in red, the metabolites whose concentration is increased in the diabetes samples (compared with values in samples from healthy controls). Molecules whose concentration was not determined are within ellipses; they are included as nodes in metabolism interconnections. Areas in color represent the main metabolic pathways. The mitochondria symbol indicates a molecule/group of molecules that may be in mitochondria or are directly/indirectly related to mitochondria.

Regarding the over-represented acyl-carnitine in diabetic patients, hydroxy-butyryl-carnitine, it should be that some acyl-carnitines provide benefits in diabetes, from improving insulin sensitivity to controlling oxidative stress (see Ref. [43] for review). However, other studies show that medium-chain acyl-carnitines mediate early progression to type 2 diabetes [44]. As there are several different acyl-carnitines and additional metabolites (see Figure 3), metabolomics appears to be an instrumental approach to determining patterns associated with diabetes risk versus patterns associated with appropriate disease management.

## 4. Materials and Methods

### 4.1. Subjects

A total of 38 samples were collected; 31 samples of healthy controls (15 men and 16 women, mean age 56, range 24–76, and seven from type 2 diabetes patients (four men and three women, mean age 72, range 65–76) were collected. None of the patients or healthy controls reported any kidney disease, and creatinine and urea plasma levels were within reference values. Axial length (AXL) was 23.92 mm (21.25–27.29 range) in the control group and 24.37 mm (23.23–25.32 range) in the diabetes group. Glycosylated hemoglobin measured one month before the surgery was within reference values (<40 mmol/mol) in the control group and <45 mmol/mol in the patient group. Three patients had controlled hypertension (43%), one hyperlipidemia (14%), two (29%) a previous episode of myocardial ischemia, and one (14%) did not have any comorbidity. Patient medication for diabetes mellitus was metformin. None of the individuals had previously undergone eye surgery. Participants were informed and, in terms of ethical standards, this study adhered to the tenets of the declaration of Helsinki. The study has been evaluated by the *Comitè d’Ètica de la Investigació de les Illes Balears (CEI-IB)*; the committee deemed the study exempt from review. All the patients with diabetes were under good control of glycemia. None of them showed any sign of diabetic retinopathy. The aqueous humor was collected at the beginning of the intervention. In both diabetes and control groups there were individuals with myopia or hyperopia but without cataract or eye pathologies; they underwent surgery for refractive lensectomy or ICL implanting (without withdrawing the crystalline lens).

Surgeries were performed in fasting conditions by J.S.N. The pupil was dilated and disinfected with 5% povidone iodine 5. Topical anesthesia was used and the surgery protocol was identical in all cases. The first side port, approximately 1 mm width, was performed using the microscope in the operating room. 100–150 µL AH was aspirated with a 27G needle. Samples were immediately transferred to 0.5 mL Eppendorf tubes and stored at −80 °C until analysis.

### 4.2. Metabolomics

The AbsoluteIDQ™ p180 Kit (Biocrates Life Sciences, Innsbruck, Austria), was used. 188 metabolites from BAs, amino acids, hexoses, phospho- and sphingolipids, and acyl-carnitines can be determined using this specific kit. Individual metabolites may be found in www.biocrates.com/products/research-products/absoluteidq-p180-kit, (accessed on 16 October 2022). Up to 30 μL of aqueous humor was plated in each well. The initial sample processing was as indicated by the manufacturer. Afterwards, derivatized samples were analyzed in the AB Sciex 6500 QTRAP MS/MS mass spectrometer (AB Sciex LLC, Framinghan, MA, USA) coupled to an Agilent 1290 Infinity UHPLC system (Agilent, Santa Clara, CA, USA). Data analysis was performed using Analyst and the MetIDQ™ 5.5 software.

### 4.3. Statistical Analysis

Univariate analysis of data was performed using two-sample *t* test, comparing one by one all those molecules whose concentration was reliably determined. Significant differences were considered when *p* < 0.001. For closer comparisons we selected those metabolites that met both criteria: log_2_ fold change (FC) > |0.4| and *p*-value < 0.001. Receiver operating curves (ROC) were made using the IBM SPSS software. Principal component analysis (PCA) was made using the R software.

## Figures and Tables

**Table 1:** Values of sensitivity, specificity, and area under the curve obtained from the ROC plots in Figure 1.

Metabolite	Sensitivity	Specificity	Cut-Off (μM)	AUC ^a^
N-Acetylornithine	1	1	1.010	1
Kynurenine	0.857	0.839	0.592	0.829
Creatinine	0.714	0.871	34.67	0.816
Total DMA	0.714	0.806	2.68	0.809
C3-DC (C4-OH)	0.714	0.871	0.012	0.809
Alanine	0.857	0.774	197.0	0.806

^a^ AUC: Area under the curve.

**Table 2:** Statistical correlations between the data from metabolites selected in the volcano plot. The Pearson coefficient is shown for pair-wise correlations. A total correlation would be defined by coefficient = 1. * False Discovery Rate (FDR)-corrected < 0.05.

N-Ac-Ornithine	0.364 *	0.587 *	0.551 *	0.408 *	0.568 *
	Kynurenine	0.366 *	0.464 *	−0.039	0.468 *
		Creatinine	0.774 *	0.414 *	0.736 *
			Total DMA	0.384 *	0.760 *
				C3-DC (C4-OH)	0.232
					Alanine

## Data Availability

The data produced in the study are in the published manuscript and/or in the supplementary material.

## Supplementary Materials

The following supporting information can be downloaded at: https://www.mdpi.com/article/10.3390/ijms232112752/s1.
