# Supplementary material for: Biogenic Amine Levels Markedly Increase in the Aqueous Humor of Individuals with Controlled Type 2 Diabetes
Source: Int J Mol Sci. 2022 Oct 22;23(21):12752. doi: 10.3390/ijms232112752 (PMC9658658; doi:10.3390/ijms232112752)
Supplement: Supplementary file 1 [file ijms-23-12752-s001.zip › Supplementary Material_Figs.pdf]

Supplementary Material

Biogenic amine levels markedly increase in the aqueous humor of individuals with controlled type 2 diabetes

Alejandro Lillo [1,2, †], Silvia Marin [3,4,5,†], Joan Serrano-Marín [6], David Bernal [7], Nicolas Binetti [6], Gemma Navarro [1,2], Marta Cascante [3,4,5], Juan Sánchez-Navés [8,‡] and Rafael Franco [1,6,9,‡,*]

1 Department of Biochemistry and Physiology. School of Pharmacy and Food Science. Universitat de Barcelona. Barcelona. Spain.
2 CiberNed. Network Center for Neurodegenerative diseases. National Spanish Health Institute Carlos III. Madrid. Spain.
3 Department of Biochemistry and Molecular Biomedicine, Faculty of Biology, Universitat de Barcelona (UB). 08028 Barcelona, Spain
4 Institute of Biomedicine of University of Barcelona (IBUB), University of Barcelona (UB), 08028 Barcelona, Spain
5 CIBEREHD. Network Center for Hepatic and Digestive Diseases. National Spanish Health Institute Carlos III (ISCIII), 28029 Madrid, Spain
6 Molecular Neurobiology laboratory. Department of Biochemistry and Molecular Biomedicine. Universitat de Barcelona. Barcelona. Spain.
7 Department of Genetics, Microbiology and Statistics. Faculty of Biology, Universitat de Barcelona (UB). 08028 Barcelona, Spain
8 Department of Ophthalmology, Ophthalmedic and I.P.O. Institute of Ophthalmology, Palma de Mallorca, Spain.
9 School of Chemistry. Universitat de Barcelona. Barcelona. Spain.
† These authors contributed equally to this work.
‡ These authors contributed equally to this work.
* Correspondence: rfranco123@gmail.com

IMPORTANT COMMENT FOR SUPPLEMENTARY TABLE S1 (supplied in a separate file)

In supplementary Table S1 (send in a separate Excel file) all concentrations are in µM, and due to issues with Microsoft Excel, all amounts are in µM units followed by "," instead of "." Same for p values. Example using a screeshot from the Excel file:

p-value CTRL VS. Diabetes	Aminoacids	Humor acuoso CTRL	Humor acuoso Diabetes
0,0001216	**Ala**	173,194	245,238
0,1465	**Arg**	96,172	84,952
0,5731	**Asn**	29,239	27,776
0,729	**Asp**	1,492	1,924
0,145	**Cit**	3,765	3,196
0,1672	**Gln**	570,011	591,429
0,07854	**Glu**	8,738	6,062
0,3294	**Gly**	9,074	11,429
0,4367	**His**	53,912	50,238

p-value for Ala is 0.0001216

Concentration for Ala in control group is 173.19 µM

Concentration for Ala in diabetes group is 245.24 µM

CONTENTS IN THIS FILE FOR SUPPLEMENTARY FIGURES

- Supplementary Figure S1.
- Supplementary Figure S2.

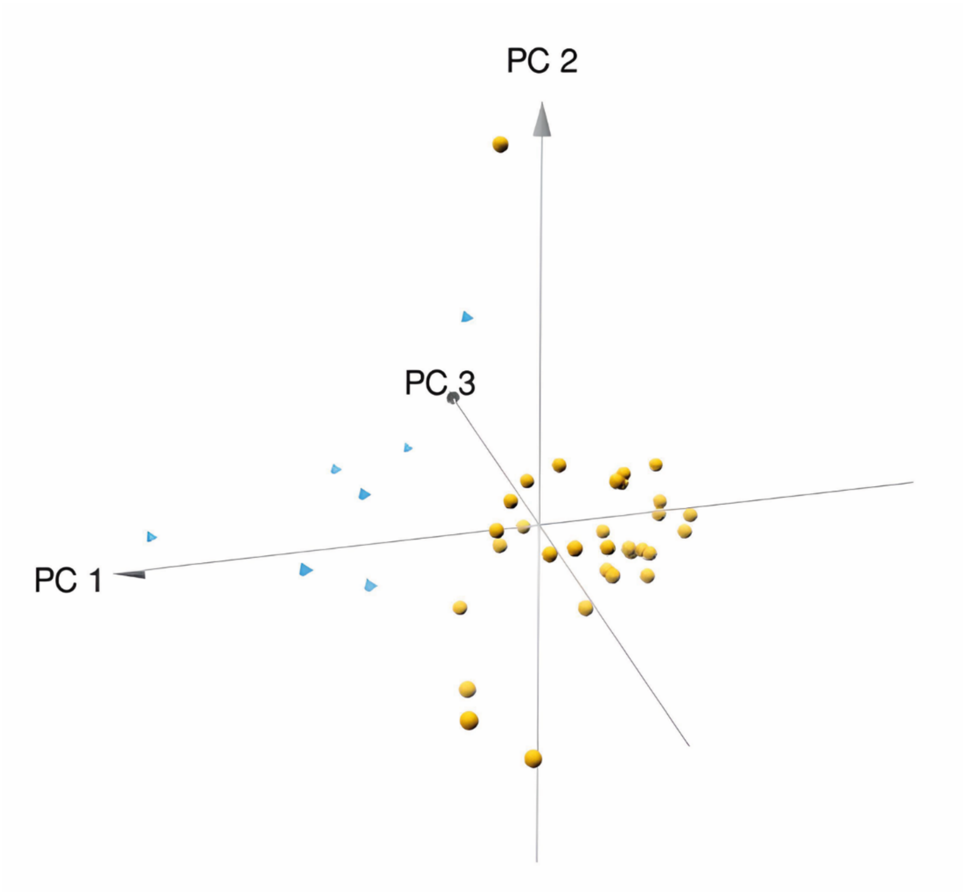

Supplementary Figure S1. Principal component analysis. The data for controls are in yellow and the data for diabetic individuals are in blue. The principal component (PC) 1, the PC2, and the PC3 have, respectively, the following eigenvalues: 2.50, 0.81 and 0.47.

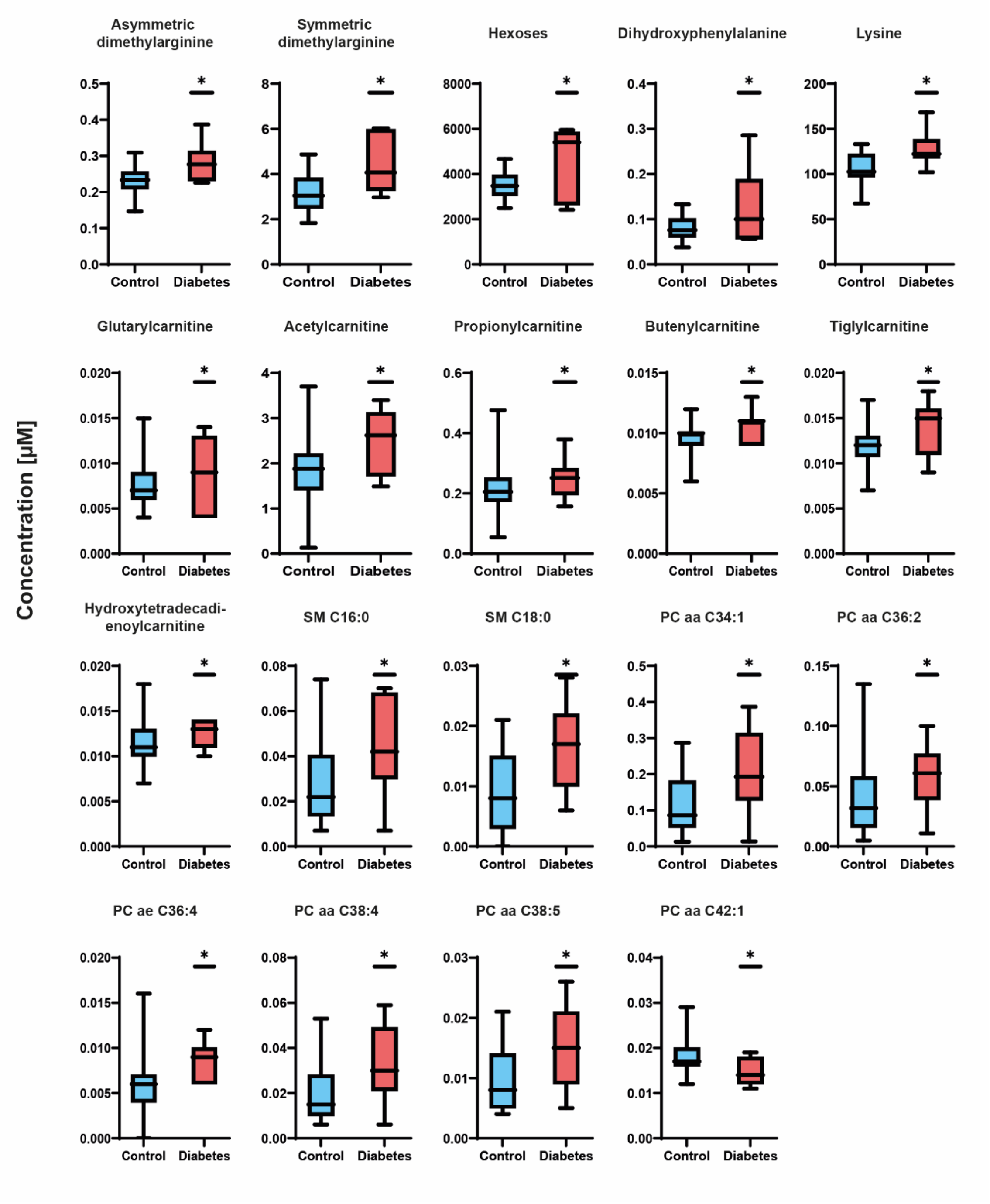

Supplementary Figure S2

Compounds whose concentration is significantly different in AH from diabetes (if compared with healthy individuals). Only data for 19 compounds are here displayed because the most relevant, **those for N-acetylornithine, kynurenine, alanine, creatinine, hydroxy-butyryl-carnitine and total dimethyl-amines are in the Figure 2 of the main manuscript**. Median in box-and-whisker plots (whiskers indicate the highest and lowest value determined for each compound in each group, control, or diabetes) * $p<0.05$ (See Methods in the main manuscript).
